# Knowledge Graphs Based on Meta-Analysis Papers Improve the Quality of Case Formulation: Mixed Methods Design

**DOI:** 10.2196/76808

**Published:** 2026-06-30

**Authors:** Kenji Yokotani, Yasumitsu Jikihara, Kohei Koiwa

**Affiliations:** 1 Graduate School of Technology, Industrial and Social Sciences, Tokushima University, 1-1, Minamijosanjima-cho, Tokushima, 770-8502, Japan, 81 88-656-7000; 2 Department of Data Science, PsychoBit Inc., Kobe, Japan; 3 Graduate School of Human Sciences, The University of Osaka, Suita, Japan; 4 Graduate School of Education, Hokkaido University of Education, Sapporo, Japan

**Keywords:** case formulation, knowledge graph, family therapy, large language model, LLM, reframing

## Abstract

**Background:**

Case formulation (CF) is a core skill for therapists; however, creating high-quality CFs requires considerable time.

**Objective:**

This study aims to demonstrate that providing a knowledge graph based on meta-analytic literature can enhance CF quality.

**Methods:**

Five groups were established, including 4 large language model groups and 1 human expert group, each generating 25 CFs based on 25 vignettes. The control group with Claude (Sonnet 3.7; Anthropic) produced 25 CFs. The personalization group served as the control group with additional personalization prompts. The knowledge graph group used a large language model that generated 25 CFs, which was provided with a meta-analysis knowledge graph. Further incorporation of additional personalization prompts then comprised the knowledge graph with personalization group. Finally, the expert group consisted of 25 CFs generated by a human expert. These 125 CFs in total were evaluated for general quality (ie, correctness, completeness, feasibility, and consistency) using a 7-point scale and 18 essential elements with binary scores (0 or 1) by another human expert. The CFs were also qualitatively analyzed.

**Results:**

The knowledge graph and knowledge graph with personalization groups scored significantly higher than the control group in terms of correctness, completeness, and feasibility. The expert group scored significantly higher on consistency than the machine-generated groups. Additionally, there was no significant difference in the feasibility scores among the knowledge graph, knowledge graph with personalization, and expert groups. The qualitative evaluation suggested that human CFs narrow the text to content that is easy for the client to read, whereas machine CFs are more likely to include expressions that are unnatural to the client.

**Conclusions:**

These results indicate that providing knowledge graphs to novice therapists increases the correctness, completeness, and feasibility of CF. Providing experienced therapists with knowledge graphs is suggested to improve the quality of their CF and mental health services.

## Introduction

Case formulation (CF), or case conceptualization, is a hypothesis collaboratively developed by the therapist and the client to address the client’s psychological difficulties [1,2]. It influences both the choice of therapeutic approach [3] and treatment outcomes [4]. CF is a core skill for therapists [5]; however, extensive training is required to develop high-quality CF [6]. CF involves not only the diagnosis of mental disorders but also the comprehensive assessment of the client’s psychological difficulties and associated risks [7]. Consequently, it is a time-consuming process, with previous research indicating that the development of a single CF requires an average of approximately 52 minutes [8]. In recent years, the automated generation of CF through a large language model (LLM) has been used as an auxiliary tool for therapists to create high-quality CF. It has been suggested that by using this CF, therapists can produce high-quality CF within a relatively short training period [1]. This study demonstrates that by incorporating the collection of meta-analysis evidence as a knowledge graph into LLM [9,10], a high-quality CF can be generated. High-quality CF could serve as an auxiliary clinical tool for therapists to develop their CF skills [11].

The theoretical model of this study is an evidence-based CF model [12], which emphasizes the incorporation of findings from previous research—that is, evidence—into CF. Evidence-based CF is currently considered the most reasonable approach [13]. Although therapists understand the importance of citing evidence, it has been pointed out that, in practice, CF is conducted without using evidence because of the time-consuming nature of literature searches [14]. However, theoretically, evidence-based assessments and CFs refine CFs and gradually improve their quality. [15].

**Figure 1.**
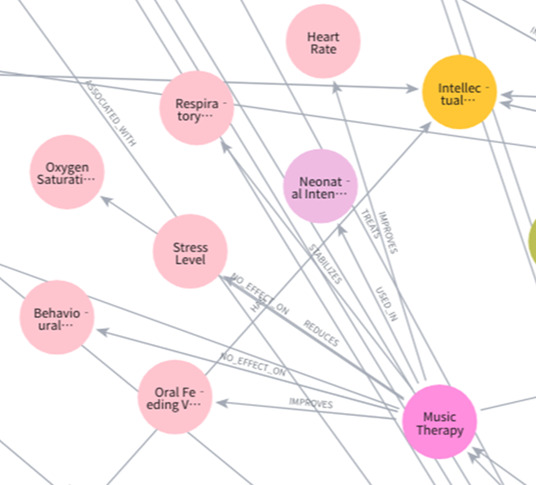
Examples of nodes and edges in a knowledge graph based on meta-analytic studies of parenting stress.

Knowledge graphs are used to retrieve evidence collections. A knowledge graph is a network that systematically connects various pieces of knowledge [16] and represents them in a graph structure (Figure 1); knowledge graphs have been constructed from clinical trials [17], medical records [18], and research papers [16]. Decision-making for therapists can be supported by such knowledge graphs [19]. Furthermore, by incorporating a knowledge graph into the LLM, more accurate diagnoses [10] and higher-quality diagnostic report generation [9] become possible.

Based on previous findings [9,10], as well as the premise that an accurate diagnosis is an essential component of high-quality CF [20], it is posited that augmenting an LLM with a knowledge graph would result in the generation of high-quality CF. Furthermore, CF is personalized through collaboration with the client [21], and personalized CF has been suggested to be more effective in treatment than nonpersonalized CF [4]. Based on these previous studies, this study also investigated the personalization of CF.

As indices of CF general quality, 4 indicators (ie, correctness, completeness, consistency, and feasibility) that were used in previous research [1] were used. In addition, as an index of the individual components of CF, 18 key components derived from previous research [2] were used to examine their presence or absence. Moreover, a qualitative evaluation was conducted to examine CFs from multiple perspectives [22]. Standardized evaluation metrics for CF were not used in this study because their reliability and validity have not yet been sufficiently established [23].

The hypotheses of this study are as follows: the LLM augmented with both the knowledge graph and personalization (knowledge graph and personalization group) is expected to generate a higher-quality CF than an LLM with no augmentation (control group). For comparison, this study also created a group with an LLM augmented with a knowledge graph only (knowledge graph–only group), a group with an LLM augmented with personalization only (personalization-only group), and a group of human therapists (expert group).

## Methods

### Construction of 25 Vignettes

Clinical psychologist A, who is the sole author of a book on CF in family therapy [24], created 25 fictional parenting stress vignettes. Various episodes were extracted from parenting blogs on *Ameba Blog*, a popular blogging platform in Japan [25]. Each episode was summarized after removing personal information and commercial content (prior confirmation was obtained from the administrators of the *Ameba Blog*, indicating there were no issues with such usage). An example of a vignette is shown in Textbox 1. Two additional fictional parenting stress vignettes were created using the same method as that used for the preliminary datasets in order to develop the evaluation criteria.

Textbox 1. **Example of a vignette.**

I’m in my 40s, raising my first son born in 2018 and my second son born in 2020 who has autism and severe intellectual disability. I'’ve been completely exhausted from caring for my second son and have been seeing a psychiatrist. Recently, when I consulted with Child Protection Services about short-stay respite care due to parental fatigue, unexpectedly my second son was taken into temporary protective custody the very next day.

Initially, I just wanted to communicate that I was at my limit with exhaustion and needed temporary childcare. However, as I explained my situation in detail, I ended up confessing that I had been so desperate that the terrifying thought, "“Would I feel relief if I killed my child...." had crossed my mind. I also mentioned that I had run out of my prescribed medication. This became a serious issue, and protective custody was decided that same day.

When I explained the situation to my husband, I thought he would be angry, but he just quietly cried. Seeing him like that, I felt overwhelmingly guilty. I realized I had caused a terrible situation.... I felt so sorry for my second son as well.

About a month after my son was taken into protective custody, discussions about ending the protection finally began. The conditions include arranging visiting nurses and helpers for me, and most importantly, securing regular short-stay respite care. However, although the city office had told us that finding short-stay facilities was the parents'’ responsibility, it turned out this should have been handled by the consultation service office. The city office’s incorrect information was one factor that led to this situation.

Currently, nearby facilities are full, and we'’re searching for short-stay options throughout the entire prefecture. I understand there may be criticism for writing this blog, but I hope my experience can be helpful to others in similar situations, so I plan to continue sharing my story.

### Construction of the Knowledge Graph

Semantic Scholar [26] was used to search for research studies related to the following 10 phrases: “maternal anxiety meta-analysis,” “parental anxiety meta-analysis,” “maternal depression meta-analysis,” “parental depression meta-analysis,” “autism spectrum disorder parenting meta-analysis,” “attention deficit hyperactivity disorder parenting meta-analysis,” “developmental disorder parenting meta-analysis,” “intellectual disabilities parenting meta-analysis,” “parenting child with chronic illness meta-analysis,” and “parenting child with chronic medical problem meta-analysis.” Up to 500 studies were retrieved per phrase, resulting in a total of 4251 studies. Subsequently, duplicate studies based on the article IDs were removed. Additionally, studies whose titles did not include both “meta” and “analysis” were excluded, resulting in 2233 selected studies. Finally, studies without available abstracts were excluded, leaving 1544 studies. The text used in the titles and abstracts of these studies (410,654 words) served as the corpus for constructing the knowledge graph.

Next, each sentence within this corpus was translated into relational data for the knowledge graph using an LLM called Claude (Sonnet 3.5; “Claude-3‐5-sonnet-20240620”; Anthropic) [27], which has been frequently used in clinical contexts [28]. The relational data in a knowledge graph explicitly indicates the relationships between entities. For example, the sentence “Parent Distress was positively associated with their Child Distress” translates into relational data as “[Parent Distress] -[:ASSOCIATED_WITH]-> [Child Distress],” illustrating 2 entities and their connecting relationship. This resulted in the extraction of 7740 entities and 2348 relationships. The constructed knowledge graph is depicted in Figure 1, where individual nodes represent entities and the edges connecting these nodes represent relationships. Knowledge graphs have also been used in clinical contexts [19].

### Generation of CFs Across 5 Groups

**Figure 2.**
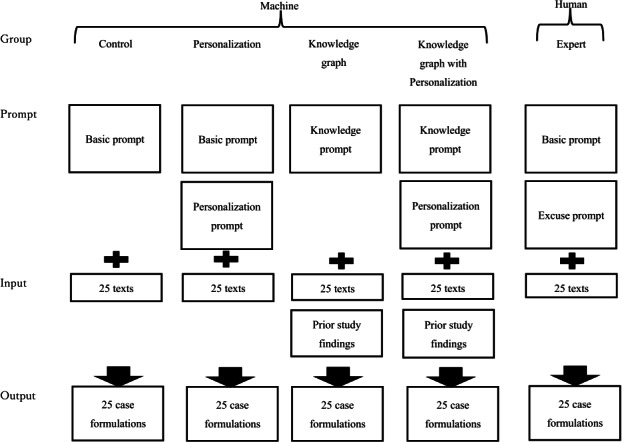
Comparison of prompts, input, and output across 5 groups.

First, for the control group, an LLM named Claude (Sonnet 3.7; “Claude-3‐7-sonnet-20250219”) [27], which has also been used in clinical contexts [29], was used with a basic prompt. It read the 25 vignettes (Textbox 1) and generated 25 CFs (Figure 2). Next, the personalization group incorporated a personalization prompt into the basic prompt; otherwise, the procedure was identical to that of the control group (Figure 2). For the knowledge graph group, the Japanese text was translated into English via Claude (Sonnet 3.7), and the previously constructed knowledge graph was queried using this translated English text. Based on prior studies [30,31], the similarity threshold was set at 0.9. Clinical psychologist A also judged the literature citations retrieved using this threshold to be appropriate. Relationships with similarity scores exceeding 0.9 between the English sentences and the relational data were extracted, compiled, and summarized as prior study findings (Table S1 in Multimedia Appendix 1). These findings were used for the knowledge graph group. Additionally, the knowledge graph group used a knowledge graph prompt alongside the 25 vignettes to generate 25 CFs (Figure 2). In the knowledge graph and personalization groups, a personalization prompt was added to the knowledge graph group’s prompt. Otherwise, the procedures matched those of the knowledge graph group. Finally, clinical psychologist B, a human expert who has presented as a symposium speaker on CF in family therapy [32], read basic prompts, excuse prompts, and 25 vignettes, and then generated 25 CFs (Figure 2). Each of the 5 groups performed 25 CFs for the 25 vignettes, producing 125 CFs (5×25).

### Evaluation of CFs

Clinical psychologist C, a Japanese expert who validated the assessment tools for stepfamilies in Japan [33], evaluated the previously created preliminary datasets (2 vignettes) across the 5 groups. These expert CFs were conducted only for the preliminary datasets by clinical psychologist A, rather than by clinical psychologist B, and were not used in the main evaluation of the 25 vignettes. The evaluation criteria consisted of 4 items assessing the general quality of CFs (on a 7-point scale) [1] and 18 items evaluating key components (presence or absence, coded as 0 or 1) [2]. Based on this evaluation, clinical psychologist A suggested adjustments to ensure broader variability in general quality scores and recommended broadly defining the presence of the 18 key components. Clinical psychologist C agreed with these recommendations. Using these criteria, clinical psychologist C evaluated the 125 CFs, which were presented randomly to ensure that clinical psychologist C remained unaware of the group assignments, thus ensuring a single-blind condition.

### Statistics

A one-factor ANOVA was performed to determine the effects of the 5 groups on the quality of the CF. The Tukey multiple comparison test was used for comparisons between groups.

### Ethical Considerations

This study did not involve experimental participants, nor did it include personal data; therefore, an ethical review of personal data was not required.

## Results

### Comparison of Text Style Among the 5 Groups

Before testing our hypotheses, we compared the length of the CF texts across the 5 groups (Table 1). As shown in Table 1, the number of characters in CFs produced by human experts was significantly lower than that of the 4 LLM conditions. Although the evaluator was blinded to the source of each CF, it is possible that the evaluator inferred human authorship based on text length. Therefore, the results should be interpreted with caution.

**Table 1.** Comparison of sentence structure, general quality, and individual key components of the 25 case formulations across the 5 groups. The significant differences in evaluation indicators were confirmed by multiple comparisons using the Tukey test.

	Control, mean (SD)	Personalization, mean (SD)	KG^a^, mean (SD)	KG_P^b^, mean (SD)	Expert, mean (SD)	Statistics	
						F test (*df*)	*P* value
Sentence structure							
Number of characters^c^	885.4 (143.1)	1002.3 (142.6)	1040.2 (135.0)	1218.8 (155.1)	542.0 (182.2)	68.01 (4, 120)	<.001^d^
General qualities							
Consistency^e^	5.36 (0.81)	5.72 (0.46)	5.64 (0.49)	5.80 (0.50)	6.20 (0.91)	5.28 (4, 120)	.001^d^
Correctness^f^	4.56 (0.77)	5.16 (0.99)	5.96 (0.73)	6.00 (0.91)	5.12 (1.01)	11.88 (4, 120)	<.001^d^
Completeness^g^	4.60 (0.76)	5.28 (0.74)	6.12 (0.60)	5.92 (0.91)	4.32 (0.90)	24.95 (4, 120)	<.001^d^
Feasibility^h^	4.76 (1.01)	5.32 (0.56)	5.48 (0.51)	5.56 (0.92)	5.12 (0.88)	4.00 (4, 120)	.004^i^
Individual key components							
List of problems	0.04 (0.20)	0.04 (0.20)	0.08 (0.28)	0.16 (0.37)	0.12 (0.33)	0.84 (4, 120)	.51
Sociocultural factors	0.04 (0.20)	0.00 (0.00)	0.00 (0.00)	0.00 (0.00)	0.00 (0.00)	1.00 (4, 120)	.41
Tailored language and metaphors	0.08 (0.28)	0.36 (0.49)	0.20 (0.41)	0.08 (0.28)	0.12 (0.33)	2.60 (4, 120)	.04^j^
Perpetuating factors	0.04 (0.20)	0.00 (0.00)	0.00 (0.00)	0.08 (0.28)	0.04 (0.20)	0.89 (4, 120)	.47
Protective factors	0.44 (0.51)	0.28 (0.46)	0.36 (0.49)	0.44 (0.51)	0.36 (0.49)	0.47 (4, 120)	.76
Personal meaning	0.16 (0.37)	0.08 (0.28)	0.08 (0.28)	0.08 (0.28)	0.04 (0.20)	0.59 (4, 120)	.67
Accessible language^k^	0.64 (0.49)	0.76 (0.44)	0.80 (0.41)	0.64 (0.49)	1.00 (0.00)	3.27 (4, 120)	.01^j^
Physiological effects	0.00 (0.00)	0.00 (0.00)	0.04 (0.20)	0.08 (0.28)	0.00 (0.00)	1.37 (4, 120)	.25
Organic causes	0.08 (0.28)	0.04 (0.20)	0.08 (0.28)	0.16 (0.37)	0.04 (0.20)	0.80 (4, 120)	.53
Recent history	0.00 (0.00)	0.00 (0.00)	0.00 (0.00)	0.00 (0.00)	0.00 (0.00)	—^l^	—
Coping strategies^m^	0.44 (0.51)	0.36 (0.49)	0.52 (0.51)	0.32 (0.48)	0.04 (0.20)	4.07 (4, 120)	.004^i^
Maintenance patterns	0.08 (0.28)	0.00 (0.00)	0.08 (0.28)	0.08 (0.28)	0.12 (0.33)	0.71 (4, 120)	.59
Relationship dynamics	0.28 (0.46)	0.20 (0.41)	0.32 (0.48)	0.28 (0.46)	0.32 (0.48)	0.29 (4, 120)	.89
Strengths and achievements	0.72 (0.46)	0.72 (0.46)	0.80 (0.41)	0.68 (0.48)	0.68 (0.48)	0.29 (4, 120)	.89
Childhood history	0.00 (0.00)	0.00 (0.00)	0.00 (0.00)	0.00 (0.00)	0.00 (0.00)	—	—
Cognitive schemas	0.04 (0.20)	0.00 (0.00)	0.00 (0.00)	0.04 (0.20)	0.08 (0.28)	0.89 (4, 120)	.47
Meaning making^n^	0.20 (0.41)	0.48 (0.51)	0.24 (0.44)	0.36 (0.49)	0.12 (0.33)	2.59 (4, 120)	.04^j^
Perceived support	0.00 (0.00)	0.00 (0.00)	0.00 (0.00)	0.04 (0.20)	0.04 (0.20)	0.75 (4, 120)	0.56

a KG: knowledge graph.

b KG_P: knowledge graph with personalization.

c “Control < expert: *P*<.001; control < KG_P: *P*=.004; expert< KG: *P*<.001; expert < KG_P: *P*<.001; expert < personalization: *P*=.006; and KG_P < personalization: *P*<.001.

d ***P<.001.

e Control < expert: *P*=.002 and KG < expert: *P*=.03.

f Control < KG: *P*<.001; control < KG_P: *P*<.001; expert < KG: *P*=.01; expert < KG_P: *P*=.006; personalization < KG: *P*=.02; and personalization < KG_P: *P*=.01.

g Control < KG: *P*<.001; control < KG_P: *P*<.001; control < personalization: *P*=.02; expert < KG: *P*<.001; expert<KG_P: *P*<.001; expert < personalization: *P*=.003; personalization < KG: *P*=.002; and personalization < KG_P: *P*=.04.

h Control < KG: *P*=.02 and control < KG_P: *P*=.005.

i **P<.01.

j *P<.05.

k Control < expert: *P*=.02 and KG_P < expert: *P*=.02.

l Not applicable.

m Expert < control: *P*=.02” and “expert < KG: *P*=.003.

n Expert < personalization: *P*=.04.

### Evaluation of the General Quality of CF

**Figure 3.**
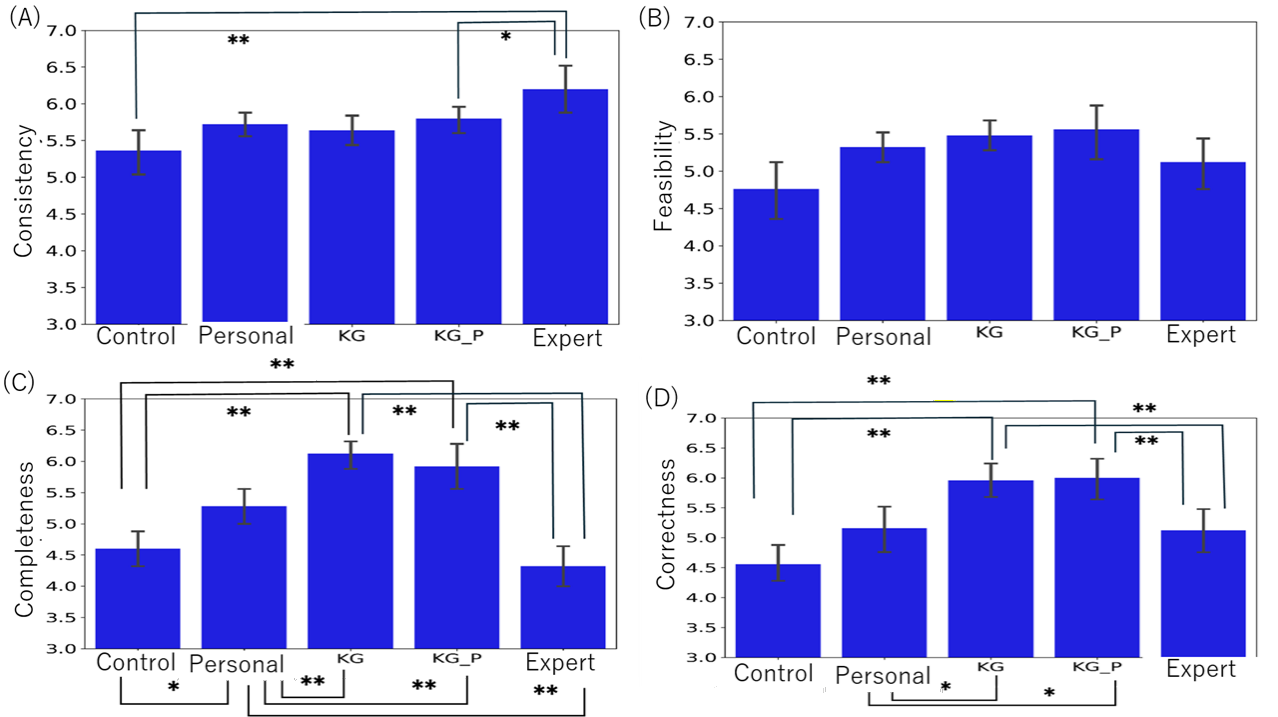
Impact of the knowledge graph (KG) on the general quality of the case formulation. (A) Consistency; (B) Feasibility; (C) Completeness; (D) Correctness. KG: knowledge gap; KG_P: Knowledge graph with personalization.

The general quality of CF was evaluated (Table 1). Regarding consistency, the expert CFs showed significantly higher scores than the machine-generated CFs (Figure 3A). Regarding correctness, both the knowledge graph group and the knowledge graph with personalization group demonstrated significantly higher scores than the control group (Figure 3D). Surprisingly, the correctness scores of the knowledge graph and knowledge graph with personalization groups were significantly higher than those of the expert group (Table 1). Similar results were confirmed for completeness, indicating that both the knowledge graph group and the knowledge graph with personalization group had significantly higher completeness scores than the control group (Figure 3C). The completeness scores of these 2 groups were also significantly higher than those of the expert group (Table 1). Regarding feasibility, the knowledge graph group and the knowledge graph with personalization group showed significantly higher scores than the control group (Figure 3B). However, there was no significant difference in feasibility between the 2 groups. These results suggest that the correctness and completeness of CF can be improved by incorporating a meta-analysis knowledge graph. Although the consistency of the machine-generated CF did not reach human expert levels, providing machines with knowledge graphs allowed the feasibility to reach levels comparable to those of experts.

### Evaluation of Key Components in CF

**Figure 4.**
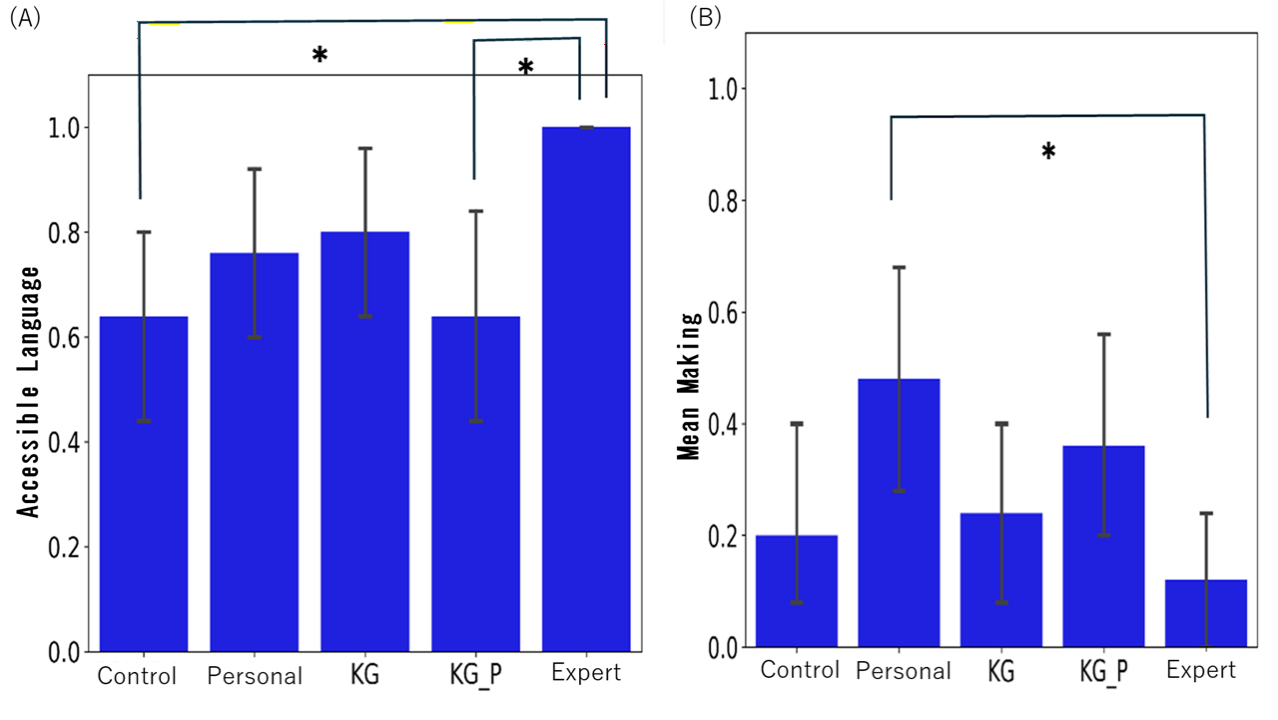
Group comparisons for accessible language (A) and mean-making (B) components in the case formulation. KG: knowledge graph; KG_P: knowledge graph with personalization. *P<.05.

The key components of CF were compared (Table 1). For the accessible language component, the expert group scored significantly higher than the control group and the knowledge graph with personalization group (Figure 4A). Regarding the mean-making component, the personalization group scored significantly higher than the expert group (Figure 4B). These findings suggest that human experts are more likely to use accessible language than machines and that personalization prompts might increase the mean-making components within the CF.

### Qualitative Analysis of CF

A qualitative analysis of the CFs from the 5 groups based on the vignette in Textbox 1 was conducted (Table 2). Concerning the use of prior study findings, citations were present in the knowledge graph and the knowledge graph with personalization groups, but absent in the control, personalization, and expert groups (Table 2). Considering that human experts typically require more time to conduct literature searches, leading to fewer citations during CF [14], it is possible that the control, personalization, and expert groups did not perform adequate literature searches during CF creation. In contrast, the knowledge graph and knowledge graph with personalization groups were provided with a list of relevant prior study findings in advance (Figure 2; Table S1 in Multimedia Appendix 1), facilitating easier use of prior study findings.

**Table 2.** Comparison of evidence use and reframing practices among the 5 groups.

	Control	Personalization	KG^a^	KG_P^b^	Expert
Use of prior study findings	None	None	“Insights from research indicates that parental stress, anxiety, and depression can also impact a child’s condition. Thus, caring for yourself is crucial for your child’s well-being as well. Parents of children with developmental disabilities are particularly prone to experiencing high stress levels, and early support has been shown to positively affect the health of both parents and children.”	“However, research clearly shows that such thoughts arise from extreme fatigue and stress, and parental stress closely relates to a child’s condition.” “Research indicates that paternal depression also affects children’s behavior.” “Research shows autism spectrum disorder (ASD) is associated with restricted and repetitive behaviors.”	None
Content reframing the client’s self-blame	“First, reaching out to the Child Guidance Center for help was a very courageous and appropriate decision. Recognizing your limits and seeking support is a sign of being a good parent. The resulting temporary protective custody may have been unexpected, but it was likely a necessary step to ensure the safety of yourself, your younger son, and your entire family.”	Raising a child with autism and severe intellectual disabilities involves unimaginable difficulties. You seem to be blaming yourself for having had the thought, ’Wouldn’t it be easier if I killed my child?’ However, please remember that this was a temporary thought arising from extreme exhaustion and overwhelming stress—it does not make you a bad parent. Rather, seeking help when these thoughts occurred was an incredibly brave action on your part.”	“You might be experiencing guilt and regret because your consultation with the Child Guidance Center unexpectedly led to temporary protective custody. However, this situation is not a ‘failure’ on your part. Rather, seeking help when you felt overwhelmed demonstrates responsible parenting.”	“Let go of the self-blame (‘It’s my fault this happened‘). Instead, recognize that your decision to seek help was an essential first step toward receiving future support.*”*	“I think it is very meaningful that you have put your experiences into words and shared them up to this point. Particularly, the thought, ‘Wouldn’t it be easier if I killed my child…?’ is something that must have been incredibly difficult for anyone to say out loud. Nevertheless, honestly expressing this was an essential SOS—a significant signal that you were hurting and needed help. The fact that you had the strength to send this SOS is extremely important.
Client expressions used during reframing	None	“Wouldn’t it be easier if I killed my child?”	“Failure”	“It’s my fault this happened”	“Wouldn’t it be easier if I killed my child…?”
Comments after response and evaluation	“While evaluating, I felt that the KG and KG_P models were highly comprehensive and accurate, and thus suitable for comparative purposes during the training process of psychologists. I believe they can be sufficiently utilized. Additionally, although some expressions appeared slightly unnatural if directly returned to the client, and these issues could be resolved if humans modify the outputs as necessary. Therefore, even for experienced psychologists, the use of these models might serve as supportive tools in preventing omissions.” (Evaluator, clinical psychologist C)				“When creating the CF, I was particularly mindful of the client’s perspective, anticipating that they would read it. Due to the significant amount of information, I was concerned whether clients could fully absorb it, so I selectively included content that I deemed manageable for the current client and omitted content that might be less accessible. Particularly in severe cases, while a diagnostic impression existed, there were instances where I was uncertain whether directly confronting the client with such information was appropriate.” (Respondent, clinical psychologist B)

a KG: Knowledge graph.

b KG_P: Knowledge graph with personalization.

The CFs were also examined from a reframing perspective. Reframing involves therapists actively attempting to transform their clients’ thoughts, which is a common technique in family therapy [34]. The vignette in Textbox 1 depicts a mother expressing self-blame, making reframing particularly applicable [35]. All 5 groups applied reframing to this aspect (Table 2). While reframing recommends directly using client phrases [36], only the expert, personalization, and knowledge graph with personalization groups adhered to this recommendation (Table 2). The control group did not use client expressions during reframing and the knowledge graph group used only single words, suggesting that they did not fully adhere to this recommendation (Table 2). This analysis indicates that personalization prompts facilitate the extraction of client-specific expressions, enhancing mean-making within CF.

Table 2 also includes comments from the experts following their responses and evaluations. The responding expert (clinical psychologist B) indicated a deliberate practice of writing only content considered easily acceptable to the recipient rather than exhaustively documenting the entirety of the CF. This practice is thought to contribute to the higher consistency observed in human-generated CFs compared to those generated by machines. Furthermore, an evaluation expert (clinical psychologist C) pointed out the unnatural expressions present in machine-generated CFs when directly returned to clients. This observation aligns with the lower scores for accessible language found in machine-generated CFs compared to human-generated CFs.

## Discussion

### Main Findings

This study demonstrates that the correctness and completeness of CF increase when using a knowledge graph based on meta-analyses. In addition, the use of a knowledge graph resulted in the feasibility of CF reaching a level comparable to that of human experts. These findings support the validity of the evidence-based CF model [12]. Incorporating the findings of meta-analytic studies into CF has 3 advantages. First, integrating academic findings enhances the accuracy of CF content [12]. Second, the inclusion of diverse academic findings increases CF completeness [15]. Third, because meta-analyses often include specific therapeutic methods, their incorporation into CF enhances feasibility [13]. This study provides concrete data to support the validity of the evidence-based CF model [12]. This study is valuable in that it enhances CF by incorporating a knowledge graph derived from meta-analytic findings into an LLM. Although previous studies have applied knowledge graphs to diagnostic assessment [30,31], research using knowledge graphs specifically for CF remains scarce. This study is novel in demonstrating that the use of a knowledge graph can improve the quality of CF. Furthermore, previous CF research has predominantly focused on cognitive-behavioral therapy in Western countries [2,23]; incorporating Asian family therapy has expanded the cultural diversity of CF research [1].

### Strengths of Human Experts

Regarding consistency, the influence of the knowledge graph was minimal, and human expert CF generally scored higher than machine-generated CF. Human experts summarized the content in paragraphs rather than bullet points, potentially contributing to their higher consistency scores. In addition, human experts included more elements of accessible language than machines. Although the machines were prompted to use simple language and adhere to these instructions, the human experts still exhibited more explicit emotional expressions. The experts effectively combined the expressions presented in the vignettes to convey nuanced meanings. Nuanced expression is a difficult area for LLMs. Although LLMs may have an advantage over the general population [37], human experts currently show an advantage, and a similar trend arises in a variety of specialties [38].

### Effects of Personalization

Personalization had a limited impact on the general quality or specific key components of CF, which is inconsistent with previous findings [4,21]. However, qualitative analysis indicated that personalization facilitates the extraction of client-specific expressions, promoting mean-making within CF. During vignette creation, writer-specific expressions were likely diminished because of the removal of proper nouns, potentially obscuring the personalization effect. Individual-specific expressions may also have been lost during the summarization process. One previous study has demonstrated that a 2-stage approach—first summarizing individual-specific characteristics and subsequently performing diagnostic reasoning—yields higher diagnostic accuracy in LLMs than direct diagnosis alone [28]. Accordingly, adopting a 2-stage approach in which individual-specific expressions are first extracted and then used to generate CFs may facilitate the development of CFs that better capture individual-specific characteristics. Future research should use longer vignettes with more writer-specific expressions and a 2-stage approach to better examine personalization effects [4].

### Practical Implications

This study has 2 practical implications. First, knowledge of graph-generated CF can be used to train novice therapists [11]. Novice therapists can compare their CFs with machine-generated CFs based on knowledge graphs, which helps them identify missing perspectives or components in their formulations [39]. Second, knowledge of graph-generated CFs can support expert therapists. Experts often lack time for literature searches, resulting in limited citations of previous research [14]. Machines can automatically extract meta-analytic literature data and present experts with current evidence, thereby improving CF quality through the timely integration of such evidence [15]. Since the knowledge extracted from meta-analyses may remain at a relatively superficial level, and even when the cited knowledge is accurate, its application in practice requires careful consideration to avoid placing undue burden on clients [30,31]. Therefore, when such knowledge is used in clinical settings, professional experience and ethical judgment are essential [28]. Indeed, the clinician involved in this study reported intentionally reducing the length of the CF to minimize the client’s burden. Because CF generation by machines is less time-consuming and more cost-effective [8], appropriately reviewing machine-generated CFs by human experts could contribute to improving the quality of CFs produced by humans.

### Limitations

This study has 4 limitations. First, there was a potential bias in the constructed data, as only 1 individual was involved in vignette creation, 1 in response, and 1 in evaluation. Because this study used only a single evaluator, interrater reliability could not be calculated. The scores used as the evaluation metric could not be examined in relation to other external measures, and thus the validity of the metric was not established. In addition, because test-retest assessments were not conducted for this measure, test-retest reliability could not be calculated. To ensure the reliability and validity of these measures, future research should incorporate test-retest evaluations and CF measures with established validity [8]. Securing multiple vignette creators, respondents, and evaluators can prevent such bias [2]. Second, human experts’ CFs differed significantly from machine-generated CFs in style and length, potentially enabling evaluators to infer human authorship despite the blinded condition. Evaluators may have identified and preferentially rated the machine responses negatively [40]. Because the CFs produced by human experts were shorter, the evaluator may have recognized them as being authored by human experts. This recognition may have contributed to the higher consistency ratings observed for the human expert condition. Future research should standardize the length and style of responses to ensure more robust blinding. Third, vignette-style presentation may not be the optimal way to present clinical cases. Presenting clinical cases in a vignette style reportedly resulted in lower diagnostic accuracy among human experts compared with presentations that list symptoms alone [41]. Similarly, when LLMs evaluate cases presented in a vignette style, they tend to generate an excessive number of diagnoses [42]. Therefore, future studies should examine the quality of CF using a format in which symptoms are presented solely as structured lists. Fourth, qualitative analysis in this study did not use thematic analysis [43]. In future research, more comprehensive qualitative analyses could be achieved by conducting thematic analysis and ensuring transparency in the coding process.

### Conclusions

Despite these limitations, our study demonstrated the effectiveness of knowledge graphs in enhancing the general quality of CF, which is consistent with studies showing the usefulness of knowledge graphs in the clinical domain [9,10,16-19]. A comparison of human and machine-generated CFs also highlighted human strengths in terms of clarity and machine strengths in terms of completeness and correctness. Future studies should demonstrate improvements in the quality of therapist-generated CFs by presenting machine-generated CFs with meta-analysis knowledge graphs to novice and expert therapists [5]. Using machines as supplementary tools to enhance CF quality will enable therapists to rapidly produce high-quality CFs [3], ultimately improving the mental health services provided to many individuals [20].

## Supplementary material

10.2196/76808 Multimedia Appendix 1 Knowledge graphs.

## Abbreviations

CF: case formulation
LLM: large language model

## References

[1] Hsieh LH, Liao WC, Liu EY (2024). Feasibility assessment of using ChatGPT for training case conceptualization skills in psychological counseling. Comput Hum Behav Artif Hum.

[2] Thrower NE, Bucci S, Morris L, Berry K (2024). The key components of a clinical psychology formulation: a consensus study. Br J Clin Psychol.

[3] Easden MH, Kazantzis N (2018). Case conceptualization research in cognitive behavior therapy: a state of the science review. J Clin Psychol.

[4] Lundkvist-Houndoumadi I, Thastum M, Hougaard E (2016). Effectiveness of an individualized case formulation–based CBT for non-responding youths with anxiety disorders. J Child Fam Stud.

[5] Kendjelic EM, Eells TD (2007). Generic psychotherapy case formulation training improves formulation quality. Psychotherapy (Chic).

[6] Dudley R, Ingham B, Sowerby K, Freeston M (2015). The utility of case formulation in treatment decision making; the effect of experience and expertise. J Behav Ther Exp Psychiatry.

[7] Mohtashemi R, Stevens J, Jackson PG, Weatherhead S (2016). Psychiatrists’ understanding and use of psychological formulation: a qualitative exploration. BJPsych Bull.

[8] Abbas MJ, Fosker H, Dudson H, Ramewal S (2025). A comparison of the quality of integrated case formulations produced by UK psychiatric trainees and an artificial intelligence-assisted application. BJPsych Bull.

[9] Gao Y, Li R, Croxford E (2025). Leveraging medical knowledge graphs into large language models for diagnosis prediction: design and application study. JMIR AI.

[10] Jia M, Duan J, Song Y, Wang J (2025). MedIKAL: integrating knowledge graphs as assistants of llms for enhanced clinical diagnosis on EMRS. Proceedings of the 31st International Conference on Computational Linguistics.

[11] Abbas M, Walton R, Johnston A, Chikoore M (2012). Evaluation of teaching an integrated case formulation approach on the quality of case formulations: randomised controlled trial. Psychiatrist.

[12] Eells TD, Lombart KG (2011). Forensic Case Formulation.

[13] Pachankis JE, Soulliard ZA, Morris F, Seager van Dyk I (2023). A model for adapting evidence-based interventions to be LGBQ-affirmative: putting minority stress principles and case conceptualization into clinical research and practice. Cogn Behav Pract.

[14] Huisman P, Kangas M (2018). Evidence-based practices in cognitive behaviour therapy (CBT) case formulation: what do practitioners believe is important, and what do they do?. Behav change.

[15] Christon LM, McLeod BD, Jensen-Doss A (2015). Evidence-based assessment meets evidence-based treatment: an approach to science-informed case conceptualization. Cogn Behav Pract.

[16] Alam F, Giglou HB, Malik KM (2023). Automated clinical knowledge graph generation framework for evidence based medicine. Expert Syst Appl.

[17] Chen Z, Peng B, Ioannidis VN, Li M, Karypis G, Ning X (2022). A knowledge graph of clinical trials ([formula: see text]). Sci Rep.

[18] Rotmensch M, Halpern Y, Tlimat A, Horng S, Sontag D (2017). Learning a health knowledge graph from electronic medical records. Sci Rep.

[19] Lyu K, Tian Y, Shang Y (2023). Causal knowledge graph construction and evaluation for clinical decision support of diabetic nephropathy. J Biomed Inform.

[20] Macneil CA, Hasty MK, Conus P, Berk M (2012). Is diagnosis enough to guide interventions in mental health? Using case formulation in clinical practice. BMC Med.

[21] van den Bergh R, Olthof M, Goldbeck F (2024). The content of personalised network–based case formulations. J Contemp Psychother.

[22] Spencer HM, Dudley R, Johnston L, Freeston MH, Turkington D, Tully S (2023). Case formulation-a vehicle for change? Exploring the impact of cognitive behavioural therapy formulation in first episode psychosis: a reflexive thematic analysis. Psychol Psychother.

[23] Flinn L, Braham L, das Nair R (2015). How reliable are case formulations? A systematic literature review. Br J Clin Psychol.

[24] Yokotani K (2022). Illustrated Case-Based Family Therapy: Systems and Narrativist Case Formulation and Intervention [Book in Japanese].

[25] Takano M, Taka F, Ogiue C, Nagata N (2024). Online harassment of Japanese celebrities and influencers. Front Psychol.

[26] Fricke S (2018). Semantic Scholar. J Med Libr Assoc.

[27] (2021). Models overview. Claude APIDocs.

[28] Kurokawa R, Ohizumi Y, Kanzawa J (2024). Diagnostic performances of Claude 3 Opus and Claude 3.5 Sonnet from patient history and key images in Radiology’s “Diagnosis Please” cases. Jpn J Radiol.

[29] Feng J, Zheng Q, Wu C M^3Builder: a multi-agent system for automated machine learning in medical imaging. https://link.springer.com/chapter/10.1007/978-3-032-06004-4_12#citeas.

[30] Gao S, Yu K, Yang Y (2025). Large language model powered knowledge graph construction for mental health exploration. Nat Commun.

[31] Lui SJ, Xiang C, Krishnaswamy S KAMEL: knowledge aware medical entity linkage to automate health insurance claims processing.

[32] Uchiyama A, Wakashima K, Koiwa K, Nagano K, Kakei K Learning brief therapy and family therapy—case formulation in brief therapy and family therapy [article in Japanese].

[33] Jikihara Y, Nozawa S, Ando S, Higginbotham BJ, Schramm DG, Adler-Baeder F (2023). Exploring the factor structure and concurrent validity of two stepfamily measures for Japanese couples. J Divorce Remarriage.

[34] Moran G, Diamond GM, Diamond GS (2005). The relational reframe and parents’ problem constructions in attachment-based family therapy. Psychother Res.

[35] Munroe M, Al-Refae M, Chan HW, Ferrari M (2022). Using self-compassion to grow in the face of trauma: the role of positive reframing and problem-focused coping strategies. Psychol Trauma.

[36] Lyddon WJ, Clay AL, Sparks CL (2001). Metaphor and change in counseling. Jour of Counseling & Develop.

[37] Sorin V, Brin D, Barash Y (2024). Large Language Models and empathy: systematic review. J Med Internet Res.

[38] Yokotani K, Takagi G, Wakashima K (2018). Advantages of virtual agents over clinical psychologists during comprehensive mental health interviews using a mixed methods design. Comput Human Behav.

[39] Liese BS, Esterline KM (2015). Concept mapping: a supervision strategy for introducing case conceptualization skills to novice therapists. Psychotherapy (Chic).

[40] Thuillard S, Adams M, Jelmini G, Schmutz S, Sonderegger A, Sauer J (2022). When humans and computers induce social stress through negative feedback: effects on performance and subjective state. Comput Human Behav.

[41] Payton EM, Graber ML, Bachiashvili V, Mehta T, Dissanayake PI, Berner ES (2024). Impact of clinical note format on diagnostic accuracy and efficiency. Health Inf Manag J.

[42] Sarma KV, Hanss KE, Halls AJM (2026). Integrating expert knowledge into large language models improves performance for psychiatric reasoning and diagnosis. Psychiatry Res.

[43] Braun V, Clarke V (2006). Using thematic analysis in psychology. Qual Res Psychol.

[44] Suchikova Y, Tsybuliak N, Teixeira da Silva JA, Nazarovets S (2026). GAIDeT (Generative AI Delegation Taxonomy): a taxonomy for humans to delegate tasks to generative artificial intelligence in scientific research and publishing. Account Res.

