# Supplementary material for: Knowledge Graphs Based on Meta-Analysis Papers Improve the Quality of Case Formulation: Mixed Methods Design
Source: JMIR Form Res. 2026 Jun 30;10:e76808. doi: 10.2196/76808 (PMC13318205; doi:10.2196/76808)
Supplement: Multimedia Appendix 1 [file formative-v10-e76808-s001.docx]

Supplementary Table 1

Examples of prior findings extracted from the meta-analysis knowledge graph

| [ Parent Distress ] -[:ASSOCIATED_WITH]-> [ Child Distress ]  [ Parent Proxy-Report ] -[:STRENGTHENS_RELATIONSHIP]-> [ Parent Distress ]  [ Depression ] -[:ASSOCIATED_WITH]-> [ Child Distress ]  [ Anxiety ] -[:ASSOCIATED_WITH]-> [ Child Distress ]  [ Parent Distress ] -[:ASSOCIATED_WITH]-> [ Child Distress ]  [ Post-Traumatic Stress ] -[:ASSOCIATED_WITH]-> [ Child Distress ]  [ Global Distress ] -[:ASSOCIATED_WITH]-> [ Child Distress ]  [ Child Self-Report ] -[:REPORTS]-> [ Child Distress ]  [ Parent Proxy-Report ] -[:STRENGTHENS_RELATIONSHIP]-> [ Child Distress ]  [ Communication Behaviours ] -[:RELATED_TO]-> [ Pediatric Emergency Care ]  [ Higher-Order Restricted And Repetitive Behaviors ] -[:ASSOCIATED_WITH]-> [ Autism Spectrum Disorder ]  [ Prenatal Maternal Stress ] -[:ASSOCIATED_WITH]-> [ Autism Spectrum Disorder ]  [ Parent Mental Health ] -[:ASSOCIATED_WITH]-> [ Pediatric Chronic Pain ]  [ Parent Mental Health ] -[:AFFECTS]-> [ Child Functioning ]  [ Depression ] -[:COMPONENT_OF]-> [ Parent Mental Health ]  [ Anxiety ] -[:COMPONENT_OF]-> [ Parent Mental Health ]  [ General Distress ] -[:COMPONENT_OF]-> [ Parent Mental Health ]  [ Parenting Stress ] -[:INFLUENCES]-> [ Child Development ]  [ Early Intervention Programs ] -[:AIMS_TO_DECREASE]-> [ Parenting Stress ]  [ Assisted Conception ] -[:ASSOCIATED_WITH]-> [ Cerebral Palsy ]  [ Fetal Movement Counting ] -[:NO_EFFECT]-> [ Maternal Worry ]  [ Parent Mental Health ] -[:ASSOCIATED_WITH]-> [ Pediatric Chronic Pain ]  [ Paternal Depression ] -[:ASSOCIATED_WITH]-> [ Child Externalizing Behaviors ]  [ Child Anxiety Symptoms ] -[:COMPONENT_OF]-> [ Child Functioning ]  [ Child Self-Report ] -[:REPORTS]-> [ Child Distress ]  [ Sleep Quality ] -[:ASSOCIATED_WITH]-> [ Perinatal Depression ]  [ Prenatal Maternal Stress ] -[:ASSOCIATED_WITH]-> [ Autism Spectrum Disorder ]  [ Prenatal Maternal Stress ] -[:ASSOCIATED_WITH]-> [ Attention-Deficit Hyperactivity Disorder ]  [ Meta-Analysis ] -[:STUDIES]-> [ Prenatal Maternal Stress ]  [ Systematic Review ] -[:STUDIES]-> [ Prenatal Maternal Stress ]  [ Child Depression Symptoms ] -[:COMPONENT_OF]-> [ Child Functioning ]  [ Paternal Bmi ] -[:AFFECTS]-> [ Offspring Health ]  [ Meta-Analysis ] -[:ANALYZES]-> [ Pediatric Chronic Illness ]  [ Pediatric Chronic Illness ] -[:CONTEXT_OF]-> [ Illness Uncertainty ]  [ Fetal Movement Counting ] -[:NO_EFFECT]-> [ Maternal Anxiety ]  [ Assisted Conception ] -[:ASSOCIATED_WITH]-> [ Autism Spectrum Disorders ] |
| --- |

Notes. This example is a list of prior study findings extracted from the vignette in Table 1.

Supplementary Table 2

Example of case formulation regarding Table 1 vignette

| Control Group |
| --- |
| "Thank you for openly sharing your feelings during such a challenging time raising your child. From reading about your experiences and emotions, I could clearly understand how difficult your daily life has been.  First, reaching out to the Child Guidance Center for help was a very courageous and appropriate decision. Recognizing your limits and seeking support is a sign of being a good parent. The resulting temporary protective custody may have been unexpected, but it was likely a necessary step to ensure the safety of yourself, your younger son, and your entire family.  Here are some suggestions to help reduce your stress, anxiety, and depression:   1. **Continue medical support:** Keep attending your psychiatric treatment and ensure you do not run out of medication. If needed, seek help from someone (such as your husband or a visiting nurse) who can assist you in managing your medication. 2. **Make full use of your support system:** Please do not hesitate to use visiting nursing services or helper services. These services are not for "weak parents," but rather they serve as essential support you rightfully deserve as a parent of a child with special needs. 3. **Strengthen cooperation with your husband:** Your husband's reaction clearly shows he understands your situation and wishes to support you. Share your emotions and difficulties honestly and try to share parenting responsibilities as much as possible. 4. **Do not blame yourself:** You mentioned several times that "this is all my fault," but this situation is no one's fault. Raising a child with disabilities is extremely challenging without professional support. Redirect the energy spent blaming yourself toward finding solutions. 5. **Take short breaks for yourself:** Try to create even short moments of personal time when your eldest son is at preschool or school, or when someone else can watch your children. Even a brief 10-minute break to drink your favorite beverage or take some deep breaths is valuable. 6. **Connect with parent associations or support groups:** Interacting with other parents facing similar situations can provide significant emotional support. Consider joining an online group to share experiences and information. 7. **Regarding securing short-term stay facilities:** It might be necessary to continue working closely with the consultation agency to search broadly for available facilities. Additionally, you may wish to contact the city's disability welfare department again to clarify and address the misinformation previously given to you.   Your decision to share your experiences through blogging could greatly support others in similar circumstances. Sharing your personal experiences might help alleviate someone else's feelings of isolation.  Please remember this: there is no such thing as a perfect parent. Parents of children with disabilities face unimaginable challenges. Asking for help is not weakness—it is a responsible choice as a parent. Step by step, on both good days and bad days, you remain an excellent parent." |
| Knowledge Graph Group |
| "# Advice for Coping with Parenting Stress  First of all, I sincerely sympathize with the difficult situation you are currently facing. Raising a child with autism and severe intellectual disabilities involves unimaginable challenges. Additionally, being honest about your feelings took significant courage.  **Regarding Your Current Feelings**  You might be experiencing guilt and regret because your consultation with the Child Guidance Center unexpectedly led to temporary protective custody. However, this situation is not a 'failure' on your part. Rather, seeking help when you felt overwhelmed demonstrates responsible parenting.  **Specific Strategies**  **1. Building a Support Network**   - **Professional Support:** Continue psychiatric treatment while actively utilizing visiting nurses and helper services. - **Family Cooperation:** Your husband’s understanding is a significant source of support. Consider discussing with your family ways to distribute the parenting responsibilities more evenly. - **Parent Training:** If there are training programs designed for parents of children with developmental disabilities, participating can equip you with practical skills.   **2. Securing Respite Care**   - **Short-term Stays:** Work closely with the city office and consultation agencies to secure regular short-term stay facilities. If facilities are fully booked, consider registering on a cancellation waitlist. - **Daytime Temporary Support:** While waiting to secure short-term stays, explore options such as short-duration daytime support services.   **3. Self-Care**   - **Medication Management:** To prevent running out of prescribed medications, consider keeping an extra supply or using a medication calendar for management. - **Small Breaks:** Even if complete rest is difficult, intentionally create short periods of relaxation for yourself. - **Avoid Self-blame:** You do not need to be a 'perfect parent.' Feeling overwhelmed occasionally is completely natural.   **Insights from Research**  Research indicates that parental stress, anxiety, and depression can also impact a child's condition. Thus, caring for yourself is crucial for your child's well-being as well. Parents of children with developmental disabilities are particularly prone to experiencing high stress levels, and early support has been shown to positively affect the health of both parents and children.  **Finally**  Your blogging could provide significant emotional support for other parents facing similar situations. By sharing your experiences, you may help someone else realize, 'You are not alone.'  Please do not blame yourself too much. Seeking help is not a sign of weakness, but rather a demonstration of parental strength and responsibility. Step by step, I sincerely hope your entire family regains a peaceful daily life." |
| Personalized Group |
| "# Advice to Alleviate Parenting Stress  First of all, I deeply respect your courage in honestly expressing your current situation and sharing it on your blog. Your experience can be a significant source of support for others struggling in similar circumstances.  **About Your Current Feelings**  Raising a child with autism and severe intellectual disabilities involves unimaginable difficulties. You seem to be blaming yourself for having had the thought, 'Wouldn't it be easier if I killed my child?' However, please remember that this was a temporary thought arising from extreme exhaustion and overwhelming stress—it does not make you a bad parent. Rather, seeking help when these thoughts occurred was an incredibly brave action on your part.  **Specific Strategies**   1. **Medication Management** To ensure you do not run out of your prescribed medication, consider creating a medication calendar with a visiting nurse or using reminder functions on your smartphone. Setting up automatic notifications when medications run low could also be very useful. 2. **Securing Short-Term Stay Facilities** It is truly unfortunate that misinformation from the city office caused confusion. Currently, the consultation agency is searching for short-term stay facilities across the entire prefecture. Although it may take some time, let's trust that a suitable facility will be found. It is also important to regularly check progress with the consultation agency. 3. **Relationship with Your Husband** Your husband’s reaction of quietly crying instead of becoming angry suggests he deeply understands and empathizes with your suffering. It is important to regularly schedule times to discuss your younger son’s care and openly share your feelings with each other. Also, consider the balance of your entire family, including caring for your eldest son born in 2018. 4. **Self-Care** Continuing psychiatric treatment demonstrates excellent self-care. Please use the time when visiting nurses or helpers are present to take care of yourself. For instance, try planning activities such as reading a favorite book, taking a bath, or talking with friends—anything that can refresh and rejuvenate you. 5. **Connecting with Parents in Similar Situations** Participating in support groups for parents raising children with autism or intellectual disabilities can offer opportunities for information exchange and emotional sharing. Online communities are also available, so it might be worthwhile to explore these.   You are not alone. Your determination to continue blogging under these challenging circumstances offers tremendous hope to other parents. Please do not blame yourself excessively; let's keep moving forward one step at a time. I sincerely hope your courageous actions lead to happiness—not only for your younger son, but also for your eldest son and yourself." |
|  |
| Knowledge graph with Personalization group |
| "# Advice for Alleviating Parenting Stress  First, I want to sincerely acknowledge the extremely difficult experience you're having with your child being placed in temporary protective custody. Raising a child with autism and severe intellectual disabilities in your 40s understandably places you under immense psychological pressure.  **Specific Support Strategies Tailored to Your Situation**  **1. Securing Professional Support**  From your text, it’s clear that securing visiting nursing, helper services, and short-term stays is currently challenging. The confusion caused by incorrect information from the city office must have been very distressing.  **Concrete Steps:**   - Proactively contact your consultation support specialist (consultation agency) and request assistance in securing short-term stay facilities. - Although the search is currently ongoing throughout the prefecture, consider also consulting agencies covering wider regions, such as regional support centers for persons with disabilities. - Utilize a medication calendar or smartphone reminders to ensure you do not run out of prescribed medications.   **2. Caring for Your Mental Health**  You seem to feel guilty about having had thoughts like, "Wouldn’t it be easier if I killed my child?" However, research clearly shows that such thoughts arise from extreme fatigue and stress, and parental stress closely relates to a child’s condition.  **Concrete Steps:**   - Continue your psychiatric treatment, and always take prescribed medications exactly as directed. - Once visiting nursing care starts, openly discuss your feelings with the nurse. - Let go of the self-blame ("It’s my fault this happened"). Instead, recognize that your decision to seek help was an essential first step towards receiving future support.   **3. Building Family Relationships**  Your husband’s reaction of quietly crying suggests he is experiencing similar emotional distress. Research indicates that paternal depression also affects children’s behavior.  **Concrete Steps:**   - Regularly schedule discussions with your husband to share each other’s feelings openly. - Provide your eldest son (born in 2018) with age-appropriate explanations and reassurance. - Create a schedule that ensures each family member has designated time for rest.   **4. Practical Daily Care Methods**  Parenting a child with autism and severe intellectual disabilities involves unique challenges. Research shows Autism Spectrum Disorder (ASD) is associated with restricted and repetitive behaviors.  **Concrete Steps:**   - Document your younger son’s behavior patterns to identify situations or times when he becomes especially distressed or confused. - Try using visual schedules or timers, which your younger son might find easier to understand. - If sensory sensitivities exist, experiment with environmental adjustments (such as reducing noise or adjusting lighting).   **5. Establishing Long-Term Support Systems**  Rather than relying solely on temporary solutions, sustainable long-term support structures are necessary.  **Concrete Steps:**   - Join parent groups or online communities for families of children with disabilities to connect with others facing similar challenges. - Writing your blog is an excellent way to express yourself. Continue sharing your experiences without fearing criticism. - In the future, consider services tailored to your younger son's growth, such as after-school day services.   You are not alone. You have the right to access support to overcome these challenging circumstances. Let’s take small steps forward to help your family regain a peaceful daily life." |
| Expert |
| I think it is very meaningful that you have put your experiences into words and shared them up to this point. Particularly, the thought, "Wouldn't it be easier if I killed my child…?" is something that must have been incredibly difficult for anyone to say out loud. Nevertheless, honestly expressing this was an essential SOS—a significant signal that you were hurting and needed help. The fact that you had the strength to send this SOS is extremely important. Although you may feel overwhelmed by guilt, your willingness to accept support clearly shows your strong desire to care for your younger son and your family. Additionally, the moment when your husband quietly cried was very striking. His response through tears rather than words may reflect a family's shared empathy and pain.  Behind this incident, it seems that multiple uncontrollable factors came together, such as running out of medication, receiving incorrect guidance from the authorities, and a shortage of available short-term stay facilities. When these environmental factors overlap, blaming yourself only makes things even harder. While it's natural to think, "I have to stay strong as a mother," what you truly need right now is to prioritize thinking, "What can I do as an individual to avoid becoming further exhausted?" Institutional supports like visiting nurses, helpers, and short-term stays are certainly important, but even more crucial is having "time when you don't have to do anything" and "a place where you can safely express your feelings and vulnerabilities." |
